# Genome analysis of a *Bacillus subtilis* strain reveals genetic mutations determining biocontrol properties

**DOI:** 10.1007/s11274-019-2625-x

**Published:** 2019-03-13

**Authors:** Bettina Bóka, László Manczinger, Sándor Kocsubé, Kadaikunnan Shine, Naiyf S. Alharbi, Jamal M. Khaled, Martin Münsterkötter, Csaba Vágvölgyi, László Kredics

**Affiliations:** 1 Department of Microbiology, Faculty of Science and Informatics, University of Szeged, Közép fasor 52, Szeged, 6726 Hungary; 2 Department of Botany and Microbiology, College of Science, King Saud University, Riyadh, 11451 Saudi Arabia; 3 Functional Genomics and Bioinformatics Group, Research Center for Forestry and Wood Industry, University of Sopron, Bajcsy-Zsilinszky u. 4, Sopron, 9401 Hungary

**Keywords:** *Bacillus subtilis*, Biocontrol, Fengycin, Genome analysis, Hypermutation, Surfactin

## Abstract

**Abstract:**

Several *Bacillus* strains are used as biocontrol agents, as they frequently have strong antagonistic effects against microbial plant pathogens. *Bacillus* strain SZMC 6179J, isolated from tomato rhizosphere, was previously shown to have excellent *in vitro* antagonistic properties against the most important fungal pathogens of tomato (*Alternaria solani, Botrytis cinerea, Phytophthora infestans* and *Sclerotinia sclerotiorum*) as well as several *Fusarium* species. Taxonomic investigations revealed that it is a member of the *B. subtilis* subsp. *subtilis* group and very closely related with the reference type strain *B. subtilis* subsp. *subtilis* 168. The sequenced genome of strain SZMC 6179J contains the genes responsible for the synthesis of the extracellular antibiotics surfactin, fengycin and bacilysin. Compared to strain 168, a prophage-like region is missing from the genome of SZMC 6179J, while there are 106 single nucleotide polymorphisms and 23 deletion-insertion polymorphisms. The high biocontrol potential of strain SZMC 6179J may results from a single base deletion in the *sfp* gene encoding the transcription factor of the surfactin and fengycin operons. Hypermutated regions reflecting short-time evolutionary processes could be detected in SZMC 6179J. The deletion-insertion polymorphism in the *sfp* gene and the detected hypermutations can be suggested as genetic determinants of biocontrol features in *B. subtilis*.

**Graphical Abstract:**

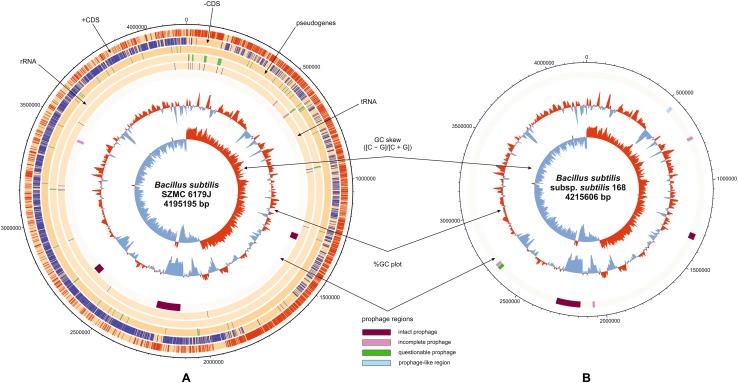

**Electronic supplementary material:**

The online version of this article (10.1007/s11274-019-2625-x) contains supplementary material, which is available to authorized users.

## Introduction

*Bacillus* strains are successful biological pest control agents by competition for nutrients and the ecological niche in the rhizosphere. They also produce various antibiotics and extracellular enzymes and induce systemic resistance mechanisms in plants (Cawoy et al. 2011, 2015; Emmert and Handelsman 1999; Jourdan et al. 2009; Kloepper et al. 2004; Shoda 2000).

Complete genome sequences are also available for some biocontrol strains of the genus *Bacillus* (Borriss 2015). Earl et al. (2012) published full genomes of four *B. subtilis* strains (*B. subtilis* subsp. *subtilis* RO-NN-1 and AUSI98, *B. subtilis* subsp. *spizizenii* TU-B-10 and DV1-B-1) and the reference type strains DV1-F-3(T) and RO-H-1(T) of the two closely related species *B. vallismortis* and *B. mojavensis*, respectively. Zeigler (2011) revealed that the genome sequence of *B. subtilis* subsp. *spizizenii* W23 shares a 3.6 Mb core genome with the intensively studied model organism *B. subtilis* subsp. *subtilis* 168 (Zeigler et al. 2008), and the gene order within this core has been strongly conserved. Additionally, the W23 genome has 157 accessory (non-core) genome segments that are not found in *B. subtilis* subsp. *subtilis* 168, while its genome has 141 segments not found in strain W23. Deng et al. (2011) presented the complete genome sequence of *B. subtilis* strain BSn5 isolated from *Amorphophallus konjac* callus tissue and showing strong inhibitory activity to *Pectobacterium carotovorum* subsp. *carotovorum*, which causes *Amorphophallus* soft rot disease. Compared with *B. subtilis* subsp. *subtilis* strain 168, 9 DNA fragments (> 5 kb) were found to be inserted and 8 DNA fragments (> 5 kb) were lost in strain BSn5. The changes affect prophage sequences, cell wall synthesis, antibiotic synthesis, sporulation regulation, mobile elements, a restriction modification system and the major facilitator superfamily MFS, which may contribute to the endophytic nature of strain BSn5. Guo et al. (2013) found large gene clusters of the rhizobacterium *B. subtilis* XF-1 that are related to the nonribosomal synthesis of antimicrobial lipopeptides and polyketides. The strain was also found to possess a gene cluster involved in the synthesis of chitosanase responsible for the suppression of the pathogen *Plasmodiophora brassicae*. Guo et al. (2014) also reported the fully annotated genome of *B. subtilis* strain BAB-1, and identified the genes encoding for active antifungal compounds in this biocontrol strain which is highly efficient against tomato gray mold. Approximately 5.2% of the genome of strain BAB-1 was found to be devoted to the synthesis of antimicrobial products, including antibiotics produced by non-ribosomal peptide synthetases (NRPSs) and polyketide synthases (PKSs), lantibiotics, as well as bacillibactin. Among these products, the lipopeptides surfactin and fengycin were also found in the strain. Fengycin was identified as a major active antifungal compound in growth inhibition of *Botrytis cinerea*. When applied in combination with fengycin, surfactin showed synergistic actions which were confirmed by antifungal assay in vivo. Luo et al. (2015) analyzed the full genome of *B. subtilis* 916, a strain highly active against filamentous fungi. This strain not only coproduces the three families of well-known lipopeptides, i.e. surfactins, bacillomycin Ls (iturin family) and fengycins, but also produces a new family of lipopeptides called locillomycins. The genome of the strain contains four NRPS gene clusters (*srf, bmy, fen* and *loc*), which are responsible for the biosynthesis of surfactins, bacillomycin Ls, fengycins and locillomycins, respectively. The complete annotated genome sequence of *B. subtilis* SG6 antagonistic to *Fusarium graminearum* has also been released (Zhao et al. 2014). The distinct *B. subtilis* strains produce more than two dozens of structurally diverse antimicrobial compounds, and 4–5% of their genomes is related with antibiotic production (Stein 2005).

During this study we performed genome analysis to identify possible genetic determinants of biocontrol features in the case of *B. subtilis* SZMC 6179J, a strain shown to exert excellent in vitro antagonistic properties against tomato pathogens (Vágvölgyi et al. 2013). A preliminary taxonomic identification, extracellular enzyme and antibiotic secretion characteristics, and a wide in vitro antagonistic spectrum of strain SZMC 6179J were reported previously (Vágvölgyi et al. 2013). Strain SZMC 6179J produces surfactins and fengycins, and it is effective against *Xanthomonas vesicatoria* and a wide set of phytopathogenic filamentous fungi (Manczinger et al. 2011; Szekeres et al. 2013; Vágvölgyi et al. 2013).

## Materials and methods

### Bacterial strain and growth conditions

Strain *B. subtilis* SZMC 6179J was isolated from tomato rhizosphere during a previous study (Vágvölgyi et al. 2013) and deposited in the Szeged Microbiology Collection (SZMC; http://www.szmc.hu), Szeged, Hungary. The strain was maintained on yeast extract-glucose (YEG) medium (yeast extract 0.2%, glucose, 0.2%, bacto agar 2%) at 25 °C.

### Genome sequencing

Strain SZMC 6179J was isolated two years before the whole genome sequencing from the rhizosphere of tomato as a single colony, possibly developing from a single spore, and maintained on YEG medium by subculturing about 50 times. As this 50-times-passed culture was subjected to full genome sequencing, the resulting reads also reflect the population genomics properties of the strain. Genome sequencing was performed by the cycled ligation sequencing on a SOLiD V4 system (Life Technologies) at Baygen (Szeged). Assembly was performed using the Genomics Workbench 4.7.2 (CLC Bio) and the Gapped SOLiD Alignment 1.2 plug-in (Omixon). Annotation was performed with the NCBI Prokaryotic Genome Annotation Pipeline (PGAAP) (http://www.ncbi.nlm.nih.gov/genomes/static/Pipeline.html). The 50-nucleotide-long reads were mapped to the annotated genome of the type strain *B. subtilis* subsp. *subtilis* 168 (GenBank ID: NC_000964) with Genomics Workbench 4.7.2. The resulting consensus sequence was used to upload the whole genome sequence of strain SZMC 6179J to the NCBI GenBank (ID: CP015004.1). The diagram showing the properties of the full genome of the strain was constructed by DNAplotter (http://www.sanger.ac.uk/science/tools/dnaplotter) (Carver et al. 2009).

### Taxonomic investigations

The exact taxonomic position of the strain was determined by multilocus sequence typing (MLST), which was successfully used for the *B. subtilis* group by Kamada et al. (2015) who used internal fragments of seven genes (*purH, glpF, pycA, ilvD, rpoD, tpiA* and *pta*) suggested by the MLST database (PubMLST; http://pubmlst.org/bsubtilis/) (Jolley and Maiden 2010). In our study, the complete sequences of nine genes (the seven previously mentioned ones as well as *gyrA* and *gyrB*) were mined from the genome of SZMC 6179J and further 173 full *Bacillus* genomes. Sequences for each gene were individually aligned by PRANK v.140603 (Löytynoja 2014) with default settings, which was followed by concatenation of the alignments with SequenceMatrix 1.8 (Vaidya et al. 2011) and partitioning of the dataset by the nine loci. Maximum likelihood (ML) inferences were generated from the dataset with raxmlGUI 1.5b1 (Silvestro and Michalak 2012) using the executables of RAxML 8.2.7 (Stamatakis 2014) under the GTR model with gamma-distributed rate heterogeneity with 1000 thorough bootstrap replicates.

### Mining of secondary metabolism-related genes and prophage cluster sequences, single nucleotide polymorphisms (SNPs) and deletion-insertion polymorphisms (DIPs)

Gene clusters of putative antimicrobials were searched for by the web-based genome mining tool antiSMASH (http://antismash.secondarymetabolites.org) (Blin et al. 2013; Medema et al. 2011; Weber et al. 2015). The presence of prophage sequences in the *Bacillus* genomes was analyzed with the PHAST search system (http://phast.wishartlab.com) (Zhou et al. 2011). The distribution of a specific prophage-like region in *Bacillus* strains was examined by BLAST (Zhang et al. 2000) and the results were visualized with the Kablammo server (Wintersinger and Wasmuth 2015). SNPs and DIPs were mined from the aligned reads with CLC Sequence Viewer v.6.5.3. and CLC Genomics Workbench 5.1.

### In vitro antifungal activity testing

Confrontation tests were carried out on YEG medium. Strain *B. subtilis* SZMC 6179J and a series of phytopathogenic fungi from the Szeged Microbiology Collection (http://www.szmc.hu) listed in Table 4 were inoculated on the surface of agar plates with 3 cm spacing. Control plates were inoculated only with the respective phytopathogenic fungus. After 5 days of incubation, the colony radii of the phytopathogenic fungi were recorded and biocontrol index (BCI) values were calculated according to the formula: BCI = (*C* − *T*)/*C* × 100, where C and T are the colony radius values of the phytopathogenic fungi in the absence and presence of the bacterium, respectively (Nene and Thapliyal 1993).

## Results

### Main characteristics of the complete genome of *Bacillus subtilis* SZMC 6179J

**Fig. 1:**
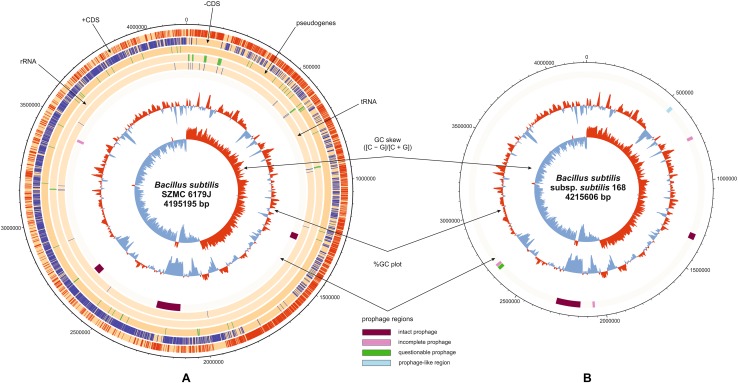
Comparison of the genomes of *B. subtilis* SZMC 6179J (**A**) and *B. subtilis* subsp. *subtilis* strain 168 (**B**). Intact, incomplete and questionable prophages are indicated according to the hits of PHAST searches. **B** also shows the prophage-like region 529,444–549,854 which is not detected by PHAST (light blue box)

**Fig. 2:**
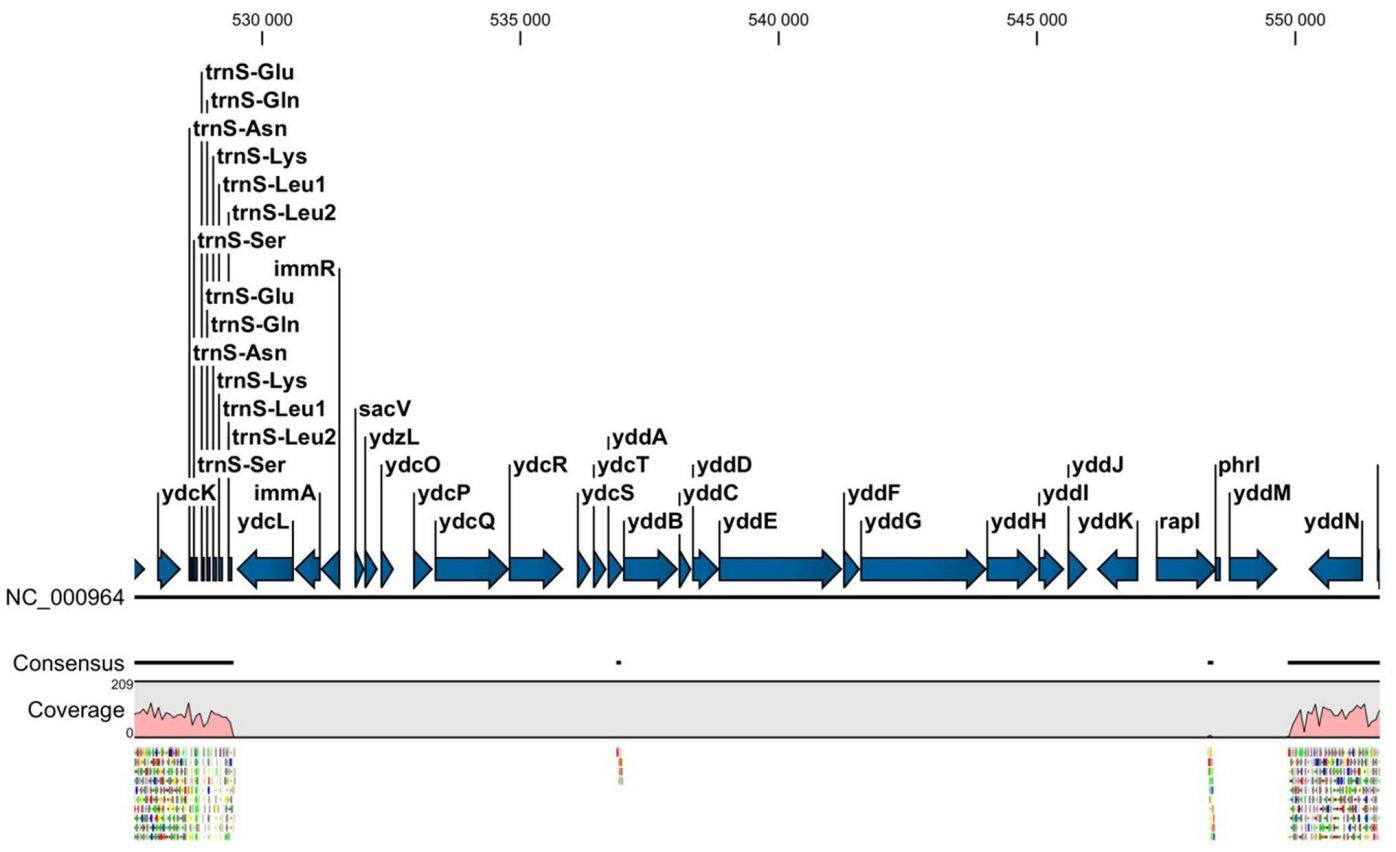
The prophage-like region of *B. subtilis* subsp. *subtilis* strain 168, which is missing from *B. subtilis* SZMC 6179J. *ydcL*: phage integrase; *immA*: immunity anti-repressor conserved in prophages; *immR*: phage element transcriptional regulator; *sacV*: transcriptional regulator with extrachromosomal origin; *ydzL, ydcO, ydcP*: hypothetical proteins; *ydcQ*: DNA wielding protein; *ydc*R: replication protein, mobile element region; *ydc*S, *ydc*T, *ydd*A-*ydd*G: hypothetical proteins; *yddH*: cell wall hydrolase, mobile element region; *yddL*: hypothetical protein; *yddJ*: lipoprotein; *yddK*: hypothetical protein; *ydd*M: helicase mobile element region; *rapL*: response regulator aspartate phosphatase; *phrL*: secreted regulator of the phosphatase

**Fig. 3:**
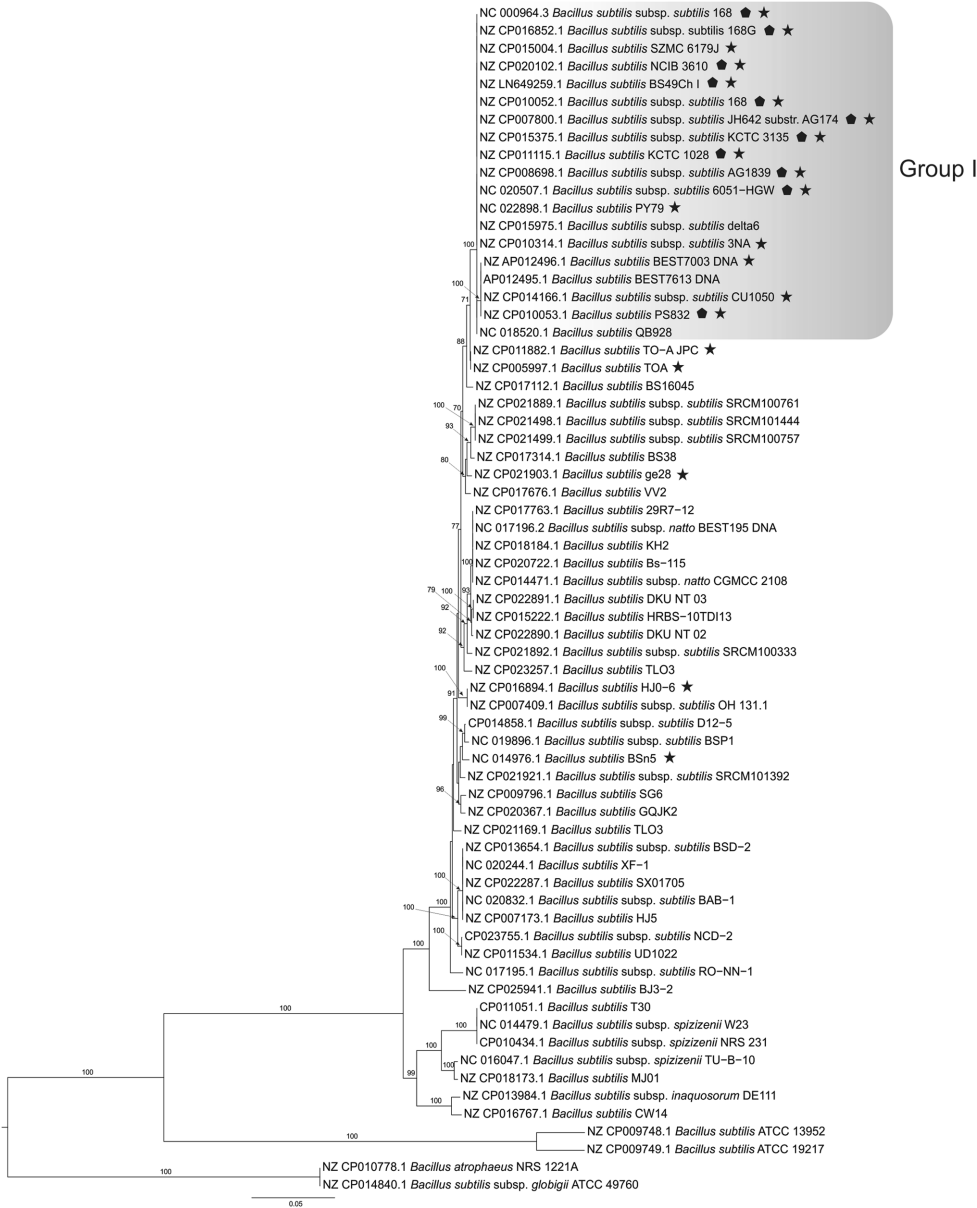
Maximum Likelihood phylogenetic tree of *Bacillus subtilis* strains constructed on the basis of nine complete gene sequences (*gyrA, gyrB, purH, glpF, pycA, ilvD, rpoD, tpiA* and *pta)* by the MLST approach. Numbers at branches indicate bootstrap values estimated by 1000 thorough bootstrap replicates under the GTR + Γ model with ten partitions. Strains containing the full prophage-like region (corresponding to 529,444–549,854 in *B. subtilis* subsp. *subtilis* strain 168) in an intact form are marked with filled pentagon. Strains containing the full, probably functional copy of the *yqcG* gene are marked with filled five pointed star. A version of the tree with a higher number of related *Bacillus* strains is shown in Online Resource Fig. 1

*Bacillus subtilis* SZMC 6179J has a single circular chromosome of 4,195,195 bp (GenBank ID: CP015004.1, Fig. 1), with a GC content of 43.6%, 4276 coding genes, 46 tRNAs, 10 rRNA loci and 13 pseudogenes (Fig. 1A). Compared to the reference genome of the type strain *B. subtilis* subsp. *subtilis* 168, large segments are missing from the genome of strain SZMC 6179J in nucleotide regions 529,444–536,858, 536,946–548,309 and 548,412–549,854. These segments are occurring within a region of the reference genome, which contains some genes of phage origin (Figs. 1B, 2), suggesting that perhaps the entire region is a prophage, or a modified prophage cluster. However, the investigation of the reference strain’s genome for the distribution of prophage sequences by PHAST (Phage Search Tool) did not reveal any prophages or prophage traces. The presence of this region was examined in other *Bacillus* strains by BLAST (Basic Local Alignment Search Tool) and the results were visualized with Kablammo, which revealed that besides *B. subtilis* subsp. *subtilis* 168, only 11 other strains have this prophage-like region in an intact form, while it is entirely missing from SZMC 6179J and a series of other *B. subtilis* strains. Strains with the full prophage-like region are present only in the “Group I” of *B. subtilis* subsp. *subtilis* (Fig. 3).

### The exact taxonomic position of *Bacillus subtilis* SZMC 6179J

Strain SZMC 6179J was identified in a previous study (Vágvölgyi et al. 2013) as *Bacillus subtilis* by the sequence analysis of a fragment of the *gyrA* gene (Genbank accession number: JX683908) according to Reva et al. (2004). Phylogenetic analysis by the MLST approach performed with the full sequences of nine genes (*gyrA, gyrB, purH, glpF, pycA, ilvD, rpoD, tpiA* and *pta*) revealed that strain SZMC 6179J is belonging to the *B. subtilis* subsp. *subtilis* group and is closely related with the type strain *B. subtilis* subsp. *subtilis* 168 (Fig. 3, Online Resource 1).

### The presence of antibiotic gene clusters in the genome of *B. subtilis* SZMC 6179J

**Fig. 4:**
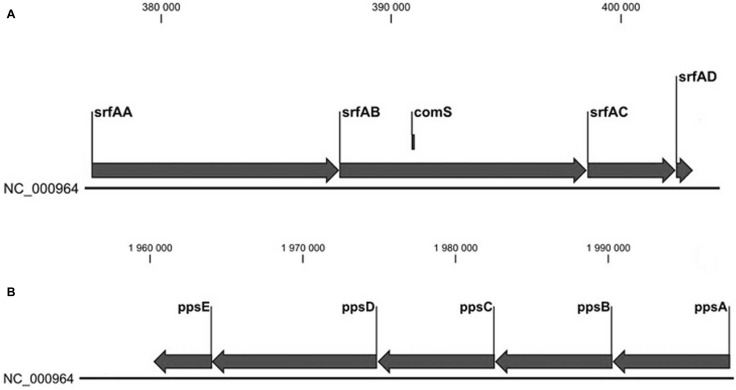
The structure of surfactin (*srfA*) (**A**) and plipastatin (= fengycin) (**B**) operons of *B. subtilis* subsp. *subtilis* str. 168. The small *comS* gene within the *srfAB* gene is a regulator gene responsible for genetical competence

Biocontrol microorganisms frequently produce distinct antibacterial and antifungal compounds. The genome of strain SZMC 6179J was analyzed for gene clusters of putative antimicrobials by the web-based genome mining tool antiSMASH (antibiotics and Secondary Metabolite Analysis Shell). The outlined clusters are presented in Table 1. We investigated the distribution of gene clusters of surfactin and fengycin in other strains of the genus *Bacillus* with the microbiological BLAST system at NCBI (National Center for Biotechnology Information) against full genomes. The structures of these operons are shown on Fig. 4. BLAST analyses with the surfactin and fengycin operons revealed that the full size surfactin operon is more abandoned in the genus than the fengycin operon. Strain SZMC 6179J contains both operons and in a potentially functional state. In the fengycin operon, large or complete deletions are frequent in other *Bacillus* strains.

**Table 1:** Results of the search for antimicrobial gene clusters in the genome of *B. subtilis* SZMC 6179J

Cluster	Type	From	To	Most similar known cluster
Cluster 1	Microcin	430	20,578	*Lactobacillus casei* (12% of genes show similarity)
Cluster 2	Microcin	20,898	41,046	*Lactobacillus* sp. (13% of genes show similarity)
Cluster 3	Microcin	81,155	107,159	*Paenibacillus polymyxa* (27% of genes show similarity)
Cluster 4	Microcin	151,511	182,147	*B. licheniformis* (30% of genes show similarity)
Cluster 5	Head-to-tail (subtilosin-like) cluster	205,275	226,765	Sporulation killing factors kfA biosynthetic gene cluster (100% of genes show similarity)
Cluster 6	NRPS	356,836	422,224	Surfactin biosynthetic gene cluster (82% of genes show similarity)
Cluster 7	Microcin	605,689	625,837	*B. pumilis* (15% of genes show similarity)
Cluster 8	Microcin	916,937	937,085	*Bacillus* sp. (16% of genes show similarity)
Cluster 9	Terpene	1,129,535	1,150,359	*B. subtilis* QB928 (58% of genes show similarity)
Cluster 10	Otherks-NRPS-Transatpks	1,748,310	1,858,136	Bacillaene biosynthetic gene cluster (92% of genes show similarity)
Cluster 11	NRPS	1,914,140	1,997,593	Fengycin biosynthetic gene cluster (100% of genes show similarity)
Cluster 12	Terpene	2,071,759	2,093,681	*B. subtilis* QB928 (47% of genes show similarity)
Cluster 13	Glycocin	2,239,136	2,259,333	Sublancin 168 biosynthetic gene cluster (66% of genes show similarity)
Cluster 14	T3pks	2,276,571	2,317,719	9 *Bacillus* genomes (100% of genes show similarity)
Cluster 15	Microcin	3,147,475	3,167,623	*Bacillus lehensis* (6% of genes show similarity)
Cluster 16	NRPS	3,240,114	3,290,023	Bacillibactin biosynthetic gene cluster (92% of genes show similarity)
Cluster 17	Other	3,563,417	3,604,172	Synthesis of pulcherriminic acid (100% of genes show similarity)
Cluster 18	Sactipeptide	3,805,918	3,827,264	Subtilosin A biosynthetic gene cluster (87% of genes show similarity)
Cluster 19	Other	3,830,263	3,871,681	Bacilysin biosynthetic gene cluster (100% of genes show similarity)

*t3pks* type III polyketide synthase cluster, *NRPS* non-ribosomal peptide synthetase

### SNPs and DIPs in comparison to the reference genome

CLC Sequence Viewer v.6.5.3 detected 106 SNPs in the genome of SZMC 6179J in comparison to the reference genome of *B. subtilis* subsp. *subtilis* strain 168. For the full list of SNPs see Online Resource 2. The data of Table 2 suggest that important functions (active genes for antibiotics and extracellular enzymes) necessary for biocontrol were not lost due to these SNPs. In the genome of strain SZMC 6179J, 23 deletion/insertion type variants (DIPs) were found; 9 out of which could be allocated to structural genes (Online Resource 3).

**Table 2:** SNPs resulting in amino acid changes in distinct protein products, possibly influencing their function

SNP by position	Name and function of the affected gene
**229,835**/Reference Position = 229,964; **Gene: cypC** Amino Acid Change = **Cys147Tyr**	Fatty-acid peroxygenase; catalyzes the alpha- and beta-hydroxylation of myristic acid in the presence of hydrogen peroxide
**284,058**/Reference Position = 284,187; **Gene: phoD** Amino Acid Change = **Asn59Lys**	Alkaline phosphatase D
**325,419**/Reference Position = 325,548 **Gene: ycgA** Amino Acid Change = **Arg70Ser**	Integral inner membrane protein
**431,730**/Reference Position = 431,866 **Gene: yclM** Amino Acid Change = **His41Arg**	Aspartokinase 3
**453,244**/Reference Position = 453,384 **Gene: ycsA** Amino Acid Change = **Ser185Arg**	Tartrate dehydrogenase/decarboxylase
**746,223**/Reference Position = 766,594 **Gene: yesS** Amino Acid Change = **Lys253Glu**	AraC family transcriptional regulator; probable transcription factor regulating the pathway responsible for rhamnogalacturonan depolymerization
**914,199**/Reference Position = 934,570 **Gene: yfhP** Amino Acidid Change = **Pro291Thr**	Uncharacterized protein; may act as a negative regulator for the transcription of *yfh*Q, *fab*L, *ssp*E and *yfh*P
**1,202,445** /Reference Position = 1,222,831 **Gene: oppC** Amino Acid Change = Trp100*	Oligopeptide transport system permease protein
**1,243,898** /Reference Position = 1,264,284 **Gene: yjcM** Amino Acid Change = **Lys216Asn**	Uncharacterized protein
**1,655,463** /Reference Position = 1,675,849 **Gene: trmD** Amino Acid Change = **His227Tyr**	tRNA (guanine-N(1)-)-methyltransferase; specifically methylates guanosine-37 in various tRNAs
**2,154,053** /Reference Position = 2,174,438 **Gene: yorO** Amino Acid Change = **Arg38Gly**	SPBc2 prophage-derived uncharacterized protein YorO
**2,195,691** /Reference Position = 2,216,076; **Gene: yopA** Amino Acid Change = Trp234*	SPBc2 prophage-derived uncharacterized protein YopA
**2,382,673** /Reference Position = 2,403,064 **Gene: gudB** Amino Acid Change = **Ala96Glu**	Cryptic catabolic NAD-specific glutamate dehydrogenase
**2,841,739** /Reference Position = 2,862,132 **Gene: radC** Amino Acid Change = **Leu135Ser**	Putative DNA repair protein
**3,078,981** /Reference Position = 3,099,373 **Gene: amyD** Amino Acid Change = **His84Leu**	Putative ABC transporter permease protein
**3,953,514** /Reference Position = 3,973,920 **Gene: cydD** Amino Acid Change = **Ser391Phe**	ATP-binding/permease protein

In strain SZMC 6179J a single nucleotide deletion in position 407,533 causes a frameshift in the *sfp* gene - encoding a phosphopantetheine-transferase-compared to strain 168 in a way that it results in a normal size Sfp. The size of the extracted gene sequence is 675 bp. After translation, a BLASTP search performed at NCBI showed that the deletion resulted in a complete amino acid sequence identical with that of the following 10 *Bacillus* Sfp proteins: *B. subtilis* subsp. *subtilis* str. NCIB 3610 (ABV89947.1), *B. subtilis* subsp. *subtilis* str. NCIB 3610 (ABV89950.1), *B. subtilis* MB73/2 (EME05049.1), *B. subtilis* subsp. *subtilis* 6051-HGW (AGG59700.1), *B. subtilis* subsp. *subtilis* (KFH30033.1), *B. subtilis* subsp. *subtilis* (KFH34853.1), *B. subtilis* KCTC 1028 (AKC45888.1), *Bacillus* sp. LM 4 − 2 (AKE22172.1), *B. subtilis* (KNB76119.1) and *B. murimartini* (KON99158.1).

### Mutation hotspots in the genome of *B. subtilis* SZMC 6179J

The strain was isolated 2 years before the whole genome sequencing, and during this time it was maintained on YEG medium by subculturing about 50 times. For genome sequencing, the DNA was extracted from this culture and not from a single cell-based culture. In this way the obtained reads reflect the genetical structure of the cell population developed through 2 years. The applied Next-Generation Sequencing method (SOLiD) produces 50-nucleotide-long reads, the number of which was 6 531 607 in the case of strain SZMC 6179J. These were aligned to the reference genome NC_000964. Considering the genome size of strain SZMC 6179J, the average coverage level was 77.84. The aligned, mapped reads were scanned for SNPs in the reads with the software CLC Genomics Workbench 5.1. at two distinct sensitivity levels (5% and 35%) to explore regions of hypermutation (e.g., a 5% sensitivity SNP scan shows the variances at nucleotide positions occurring with at least 5% frequency in the overlapping read sequences).

**Fig. 5:**
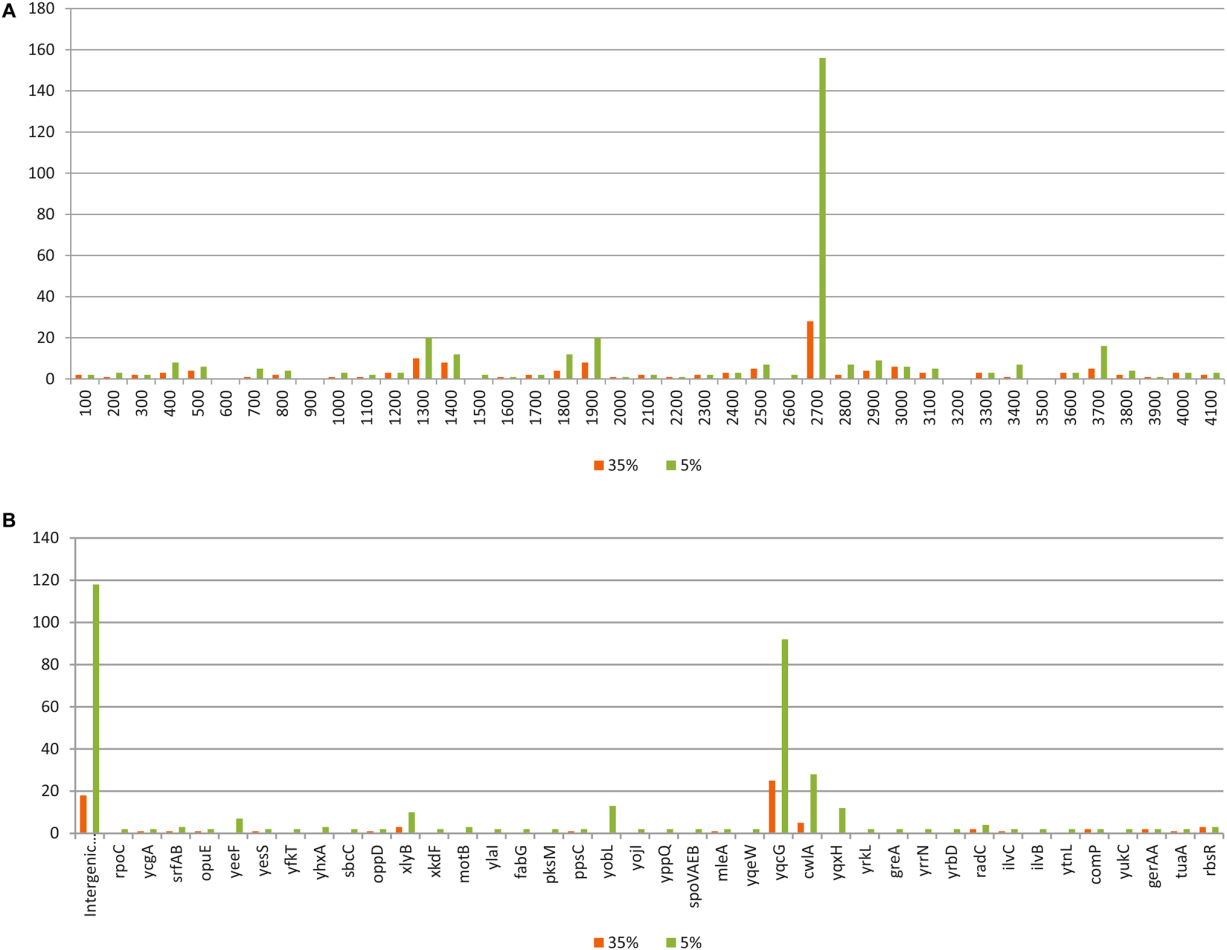
Distribution of SNPs in the SZMC 6179J cell population. **A** Distribution of SNPs in the full genome. **B** Frequency of SNPs in intergenic regions and in distinct genes in the genome population of *B. subtilis* strain SZMC 6179J as revealed by SNP scans at two distinct sensitivities (5% and 35%) in the aligned reads produced by SOLiD® Next-Generation Sequencing. Only those genes are indicated which contain at least two SNPs at 5% sensitivity scan

**Fig. 6:**
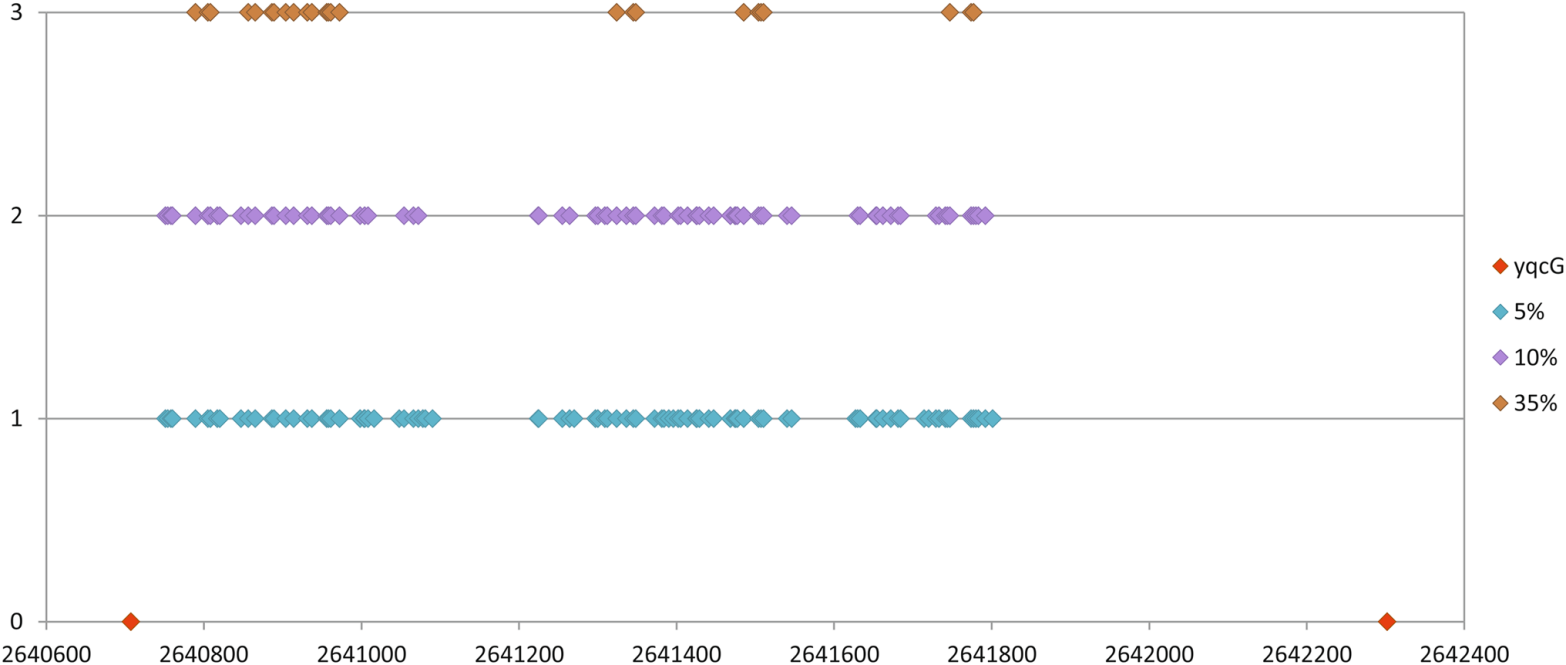
The frequency and distribution by positions of SNPs within the *yqcG* gene in the cell population of the SZMC 6179J strain, as revealed by three distinct scans in the aligned reads with different sensitivity (5, 20 and 35%). The symbols on the x axis show the positions of the start and stop codons of the *yqcG* gene

Our scan results showed that SNPs are not equally distributed on the chromosome in the genome population from the SZMC 6179J culture (Fig. 5). The most allelic variants within the cell population of strain SZMC 6179J could be found in the *yqcG* gene, which encodes for a toxic ribonuclease. Out of the 25 SNPs detected by the 35% sensitivity scan, only 5 result in amino acid changes, thus it is quite interesting, why the other 20 SNPs are so frequent in this gene, and what can possibly be the selection advantage provided by the SNPs present in this gene. The distribution of SNPs within the *yqcG* gene proved to be non-random, showing three hotspots within the N-terminal region (Fig. 6), which result in frequent amino acid changes in the sequence of the toxin (Table 3). The abundance of the *yqcG* gene in the *Bacillus* genus was also investigated by nucleotide BLAST against full genomes. The full gene is present only in some strains in the genus, mainly in the near relatives of *B. subtilis* subsp. *subtilis* str. 168 (Fig. 3). It is very interesting that a few, more distant relatives of strain 168 (e.g. strains ge28, HJ0-6 or BSn5; Fig. 3) also contain the full gene without any SNPs, suggesting the possibility of horizontal gene transfer of *yqcG* within the *B. subtilis* group.

**Table 3:** Amino acid changes in the sequence of the YqcG toxin in the cell population of strain SZMC 6179J due to the sequence variability in the coding gene

SNP scanning sensitivity	5%	20%	35%
Amino acid changes in the YqcG protein	Lys15Asn Gln18Lys Val28Ala Phe84Ile Asn89Ser Thr98Ser Ala101Glu Leu104Phe Met122Ile Asp128Glu Gly173Ala Arg240Lys Ser257Thr Ala260Val Ala316Gly Gly319Val Lys343Gln	Gln18Lys Val28Ala Phe84Ile Asn89Ser Thr98Ser Ser257Thr Ala260Val Ala316Gly Gly319Val Lys343Gln	Val28Ala Phe84Ile Asn89Ser Thr98Ser Ala260Val

Besides *yqcG*, the *cwlA* gene contains the most SNPs, while the number of SNPs is also outstanding in the *yobL, yqxH* and *xlyB* genes.

### In vitro antifungal activities of *Bacillus subtilis* SZMC 6179J

Strain *Bacillus subtilis* SZMC 6179J showed the strongest inhibition effect against *Armillaria ostoyae*, followed by *Botrytis cinerea, Bipolaris bicolor* and *Armillaria gallica* (Table 4). Furthermore, the observed BCI values of strain SZMC 6179J were also above 30% against the tested *Armillaria mellea, Alternaria alternata, Colletotrichum gloeosporioides, Curvularia spicifera, Fusarium culmorum, Phoma cucurbitacearum* and *Sclerotinia sclerotiorum* strains.

**Table 4:** Biocontrol Index values of *Bacillus subtilis* SZMC 6179J against plant pathogenic fungi

Plant pathogenic fungus	BCI
*Alternaria alternata* SZMC 16,085	32.00 ± 10.58
*Alternaria solani* SZMC 6241J	22.92 ± 9.55
*Armillaria gallica* SZMC 24,095	63.46 ± 8.81
*Armillaria mellea* SZMC 24,132	55.00 ± 13.22
*Armillaria ostoyae* SZMC 24,129	75.00 ± 5.00
*Bipolaris bicolor* SZMC 13,055	64.44 ± 3.85
*Botrytis cinerea* SZMC14526	66.67 ± 0.00
*Colletotrichum gloeosporioides* SZMC 16,086	42.31 ± 10.18
*Curvularia spicifera* SZMC 13,060	56.67 ± 3.33
*Fusarium culmorum* SZMC 11,039	44.44 ± 3.85
*Fusarium graminearum* SZMC 11,030	18.89 ± 7.70
*Fusarium moniliforme* SZMC 11,046	26.67 ± 14.05
*Fusarium oxysporum* SZMC 6237J	27.27 ± 7.87
*Fusarium solani* SZMC 11057F	20.29 ± 12.55
*Phoma cucurbitacearum* SZMC 16,088	34.67 ± 4.62
*Phytophthora infestans* SZMC 6246J	27.78 ± 5.09
*Rhizoctonia solani* SZMC 21,048	20.37 ± 3.21
*Sclerotinia sclerotiorum* SZMC 6250J	35.56 ± 13.47
*Trichoderma aggressivum* f. *europaeum* SZMC 1746	21.11 ± 10.72

*SZMC* Szeged Microbiology Collection (http://www.szmc.hu)

## Discussion

Fully annotated genomes of *Bacillus* strains with biocontrol capabilities are important tools to understand their properties, complexity, plasticity and evolution. The genome of SZMC 6179J is closely related with the reference type strain *B. subtilis* subsp. *subtilis* str. 168—a tryptophan-requiring auxotrophic strain widely used in academic research, which was isolated from *B. subtilis* subsp. *subtilis* Marburg after X-ray mutagenesis (Burkholder and Giles 1947; Zeigler et al. 2008)—the main difference between them is the lack of a prophage gene set in SZMC 6179J, as well as 106 SNPs and 23 smaller size DIPs.

The prophage-like region present in the reference genome of the type strain *B. subtilis* subsp. *subtilis* 168 but missing from the genome of strain SZMC 6179J contains many open reading frames (ORFs) with unknown function, therefore, it may be assumed that this part of the genome could be a very ancient prophage region, or traces of a phage which is not included in the recent phage sequence databases. In the publication about the full genome of *B. subtilis* subsp. *subtilis* strain 168 (Kunst et al. 1997), this region is designated as a suspected prophage-like region due to some phage elements and high AT content.

Out of the 106 SNPs detected, 18 are located in intergenic regions. Some of these SNPs might have strong influence on the regulation networks of the bacterium, thereby affecting its competitive abilities and the intensity of antibiotic secretion. From the remaining SNPs, 46 result in base substitutions within coding regions of genes, but without any amino acid changes in their resulting protein products. Finally, 42 SNPs result in amino acid changes in their corresponding gene product. Sixteen of these are changes of neutral to basic, basic to neutral, neutral to acidic, acidic to neutral, small to large or large to small amino acid, which may influence the function of the given protein (Table 1). The depsipeptide gene clusters enabling the production of surfactins and fengycins are present in the genome of *B. subtilis* SZMC 6179J. Although the presence of a given gene cluster in the genome does not necessarily mean the synthesis of the corresponding antibiotic, the effective production of two antibiotics very important for antimicrobial effectiveness, the antibacterial surfactin and the antifungal fengycin, were proved earlier in the case of strain SZMC 6179J (Bóka et al. 2016; Manczinger et al. 2011; Vágvölgyi et al. 2013). The explored SNPs and DIPs do not disturb the efficient expression of these gene clusters, as the fengycins and surfactins are produced by the strain. The production of surfactins is mediated by the *srf*A operon in *B. subtilis*, which is consisting of four genes, *srf*A-A, *srf*A-B, *srf*A-C and *srf*A-D. The plipastatin (= fengycin) operon (*pps*) consists of five genes: *pps*A, *pps*B, *pps*C, *pps*D and *pps*E (Fig. 4). These two operons encode the nonribosomal peptide synthetase (NRPS) subunits which catalyze the incorporation of amino acids into surfactin and plipastatin (Marahiel et al. 1997; Peypoux et al.^1999^). The genetic locus *sfp*, encoding a phosphopantetheine-transferase, is obligately necessary for lipopeptide production, as it converts the NRPS from the inactive apo form to the active holo form (Nakano et al. 1988; Quadri et al. 1998). The reference strain *B. subtilis* subsp. *subtilis* 168 contains the surfactin (*srf*) and plipastatin (*pps*) operons but is unable to produce these lipopeptides due to the lack of a functional *sfp* gene (Nakano et al. 1988). Although the sequence of the gene is complete, it contains an internal stop codon resulting in a truncated protein product, consequently an inactive phosphopantetheine transferase is produced, which also seems to be associated with the lack of antifungal properties in the case of this strain (Couette et al. 2010). When the wrong *sfp* was changed for a correct copy from a surfactin-producer strain of *B. subtilis*, both surfactin and fengycin production were intensively expressed in the transgenic line of strain 168 (Coutte et al. 2010). Our study revealed that a single base deletion in the *sfp* gene enables strain SZMC 6179J to produce active phosphopantetheine transferase and allows the production of the lipopeptides fengycin and surfactin, which was previously proved by TLC and HPLC investigations (Bóka et al. 2016; Vágvölgyi et al. 2013). This single base deletion in the *sfp* gene also makes strain SZMC 6179J a potential biocontrol candidate with good antifungal properties (Table 4).

Investigation of SNPs also reflects the variability within the cell population at genome sequence level, shows the regions of hypermutation in the genome where the mutations occur with an elevated frequency, and is suitable for the investigation of short-time evolution events (Brown et al. 2011; Waters et al. 2015). It has been suggested that the local hypermutation phenomenon in *B. subtilis* is in connection with the transcription-associated stationary-phase mutagenesis, which is in relation with the high expression level of the target gene and Mfd, the transcription repair coupling factor (Pybus et al. 2010; Robleto et al. 2012). Possibly this mechanism works in the affected genes of strain SZMC 6179J, at least we do not know about any other system which could produce such a high frequency of local hypermutations in *B. subtilis*. Regions of hypermutation detected in the genome of *B. subtilis* strain SZMC 6179J suggest that the fastest evolutionary events happen in genes important for competition processes and cell wall lysis. The mostly affected gene in strain SZMC 6179J was found to be the *yqcG* gene encoding for a toxic ribonuclease, which is related with the successful competition through a contact-dependent growth inhibition (CDI) of other members from the genus *Bacillus*. The product of this gene is the toxic component of a toxin-antitoxin (TA) module. The C-terminus (residues 379–531) has RNase activity and inhibits growth upon expression in *E. coli.* In vitro RNase activity and in vivo growth inhibition are neutralized by cognate antitoxin YqcF, but not by antitoxins specific to other toxins with the LXG toxin domain (Holberger et al. 2012). The N-terminal region of the toxin is responsible for the secretion and delivery of the toxin into the target competitor bacterial cells. It is supposed that this N-terminal sequence binds specifically to some membrane proteins of the target cell and these proteins are exploited for toxin entry into the cytoplasm (Willett et al. 2015). So if the bacterium population secretes a higher N-terminal sequence variety of the toxin YqcG, it will be able to suppress a higher number of distinct competitor bacteria (with distinct membrane protein surfaces). This perhaps might explain why the sequence variability is so high in this gene in the cell population of strain SZMC 6179J (Fig. 6). On the other hand, there is not even a single SNP variation in the C-terminal region where the RNase domain is located.

Further genes in strain SZMC 6179J affected by hypermutations included *cwlA, yobL, yqxH* and *xlyB*. The *cwlA* gene encodes for an N-acetylmuramyl-L-alanine amidase (Kuroda et al. 1991) with a key role in the cell wall autolysis during the sporulation processes. The YobL-CT, YxiD-CT and YqcG-CT domains from *B. subtilis* 168 have cytotoxic RNase activities, which are neutralized by the binding of cognate YobK, YxxD and YqcF antitoxin proteins, respectively. So the product of the *yobL* gene has functions like the intensively changing *yqcG* gene and is also important in the CDI systems which could be utilized by the strain during the successful habitat-occupying competition processes (Holberger et al. 2012). The product of *yqxH* is the toxic protein holin of bacteriophage origin. The holin homologue YqxH is encoded by a prophage-like sequence. Such holins can form pores in the membrane, through which the lytic enzymes of bacteriophages, which usually lack a signal peptide, gain access to the cell wall (Young and Bläsi 1995). Similarly to *cwlA, xlyB* is also an autolysin gene of prophage origin and its product is also an N-acetylmuramoyl-L-alanine amidase (Krogh et al. 1998). Previous studies could also show quick evolutionary events in *B. subtilis* strains, however, other genes were affected (Brown et al. 2011; Waters et al. 2015).

## Electronic supplementary material

Below is the link to the electronic supplementary material.

Online Resource 1. Maximum Likelihood phylogenetic tree of *Bacillus* strains constructed on the basis of nine genes (*gyrA, gyrB, purH, glpF, pycA, ilvD, rpoD, tpiA * and *pta*) by the multilocus sequence typing (MLST) approach. Numbers below branches indicate bootstrap values estimated by 1000 thorough bootstrap replicates under the GTR + Γ model with ten partitions. (DOCX 63 KB)

Online Resource 2. Full list of SNPs by position detected at 35% sensitivity scan in the genome of strain SZMC 6179J in comparison to the reference genome *B. subtilis* subsp. *subtilis* str. 168 (NC_000964.1). (DOCX 63 KB)

Online Resource 3. Deletion/insertion type variants in the genome of *Bacillus subtilis* SZMC 6179J. (DOCX 14 KB)

## Data Availability

The full genome sequence of *Bacillus subtilis* SZMC 6179J was made available in the GenBank database (Accession No: CP015004.1).
