# Supplementary figures and images for: Genome analysis of a Bacillus subtilis strain reveals genetic mutations determining biocontrol properties
Source: World J Microbiol Biotechnol. 2019 Mar 13;35(3):52. doi: 10.1007/s11274-019-2625-x (PMC6435635; doi:10.1007/s11274-019-2625-x)

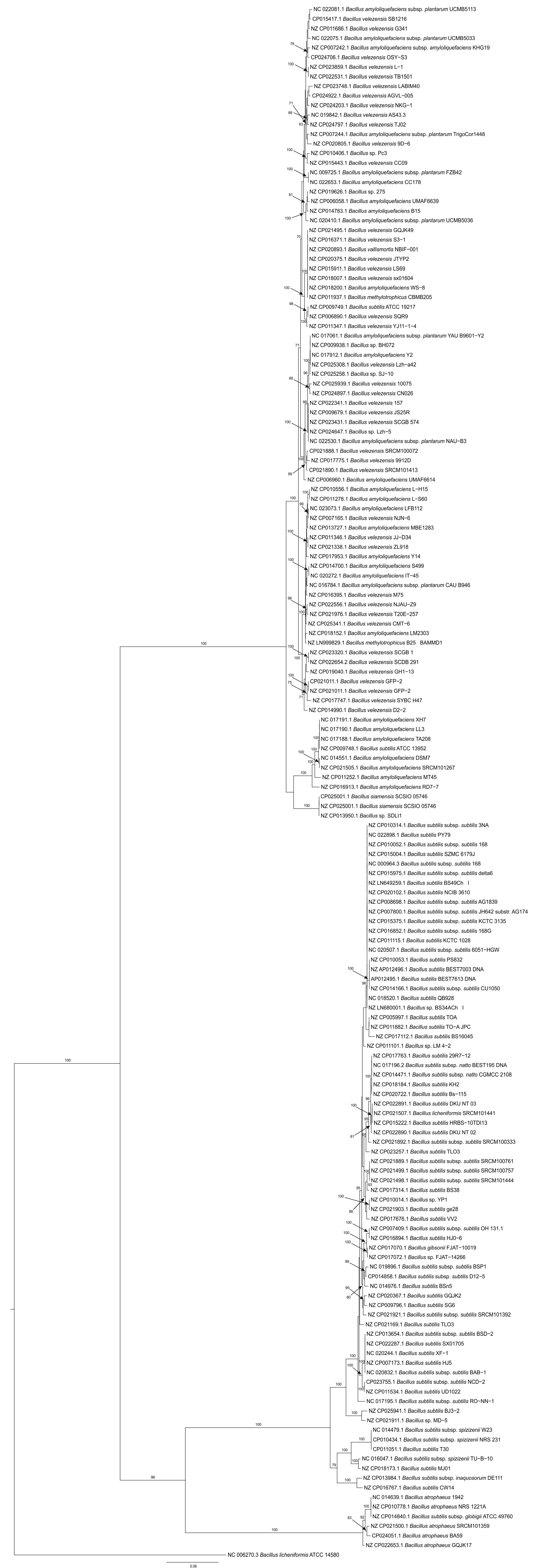

Supplement: Supplementary file 1 — Online Resource 1. Maximum Likelihood phylogenetic tree of Bacillus strains constructed on the basis of nine genes (gyrA, gyrB, purH, glpF, pycA, ilvD, rpoD, tpiA and pta) by the multilocus sequence typing (MLST) approach. Numbers below branches indicate bootstrap values estimated by 1000 thorough bootstrap replicates under the GTR + Γ model with ten partitions. (DOCX 63 KB) [file 11274_2019_2625_MOESM1_ESM.jpg]
