# Supplementary material for: Genome analysis of a Bacillus subtilis strain reveals genetic mutations determining biocontrol properties
Source: World J Microbiol Biotechnol. 2019 Mar 13;35(3):52. doi: 10.1007/s11274-019-2625-x (PMC6435635; doi:10.1007/s11274-019-2625-x)
Supplement: Supplementary file 2 — Online Resource 2. Full list of SNPs by position detected at 35% sensitivity scan in the genome of strain SZMC 6179J in comparison to the reference genome B. subtilis subsp. subtilis str. 168 (NC_000964.1). (DOCX 63 KB) [file 11274_2019_2625_MOESM2_ESM.docx]

**Online Resource 2.** Full list of SNPs by position detected at 35% sensitivity scan in the genome of strain SZMC 6179J in comparison to the reference genome *B. subtilis* subsp*. subtilis* str. 168 (NC_000964.1)

(black: intergenic, green: ingene without amino acid change, blue: amino acid change, but possibly without any strong change in function, red: amino acid change possibly with strong change in function of the protein product)

**56054** /Reference Position=56046; /Consensus Position=56054; /Reference=A; /Variation

Type=SNP; /Allele Variations=G; /Frequencies=100.0; /Counts=99; /Coverage=99;

/Overlapping Annotations=**Gene: spoVG**, CDS: spoVG; /Amino Acid Change=**Thr61Ala**

**74276** /Reference Position=74268; /Consensus Position=74276; /Reference=C; /Variation

Type=SNP; /Allele Variations=T; /Frequencies=100.0; /Counts=94; /Coverage=94;

/Overlapping Annotations=**Gene: yabT**, CDS: yabT

**116957** /Reference Position=116949; /Consensus Position=116957; /Reference=C;

/Variation Type=SNP; /Allele Variations=T; /Frequencies=100.0; /Counts=129;

/Coverage=129; /Overlapping Annotations=**Gene: sigH**, CDS: sigH; /Amino Acid

Change=**Ala117Val**

**165756^165757** /Reference Position=165749; /Consensus Position=165757;

/Reference=C; /Variation Type=SNP; /Allele Variations=T; /Frequencies=80.0; /Counts=4;

/Coverage=5

**165756^165757** /Reference Position=165750; /Consensus Position=165757;

/Reference=T; /Variation Type=SNP; /Allele Variations=A; /Frequencies=100.0; /Counts=3;

/Coverage=3

**165757** /Reference Position=165751; /Consensus Position=165757; /Reference=C;

/Variation Type=SNP; /Allele Variations=T; /Frequencies=100.0; /Counts=90; /Coverage=90

**229835** /Reference Position=229964; /Consensus Position=229835; /Reference=G;

/Variation Type=SNP; /Allele Variations=A; /Frequencies=100.0; /Counts=97;

/Coverage=97; /Overlapping Annotations=**Gene: cypC**, CDS: cypC; /Amino Acid

Change=**Cys147Tyr**

**284058** /Reference Position=284187; /Consensus Position=284058; /Reference=C;

/Variation Type=SNP; /Allele Variations=A; /Frequencies=98.1; /Counts=52; /Coverage=53;

/Overlapping Annotations=**Gene: phoD**, CDS: phoD; /Amino Acid Change=**Asn59Lys**

**325419** /Reference Position=325548; /Consensus Position=325419; /Reference=A;

/Variation Type=SNP; /Allele Variations=C; /Frequencies=100.0; /Counts=83; /Coverage=83;

/Overlapping Annotations=**Gene: ycgA**, CDS: ycgA; /Amino Acid Change=**Arg70Ser**

**394916** /Reference Position=395048; /Consensus Position=394916; /Reference=A;

/Variation Type=SNP; /Allele Variations=G; /Frequencies=100.0; /Counts=95;

/Coverage=95; /Overlapping Annotations=**Gene: srfAB**, CDS: srfAB

**407532^407533** /Reference Position=407668; /Consensus Position=407533;

/Reference=T; /Variation Type=Complex SNP; /Allele Variations=T/C;

/Frequencies=55.6/44.4; /Counts=5/4; /Coverage=9; /Overlapping Annotations=**Gene: sfp**, Misc. feature: Evidence 1a:_Function experimentally demonstrated in the studied strain; PubMedId:_10568751,_17376553,2

848009,_9384377; Product type e:_enzyme

**431730** /Reference Position=431866; /Consensus Position=431730; /Reference=T;

/Variation Type=SNP; /Allele Variations=C; /Frequencies=100.0; /Counts=79; /Coverage=79;

/Overlapping Annotations=**Gene: yclM**, CDS: yclM; /Amino Acid Change=**His41Arg**

**453244** /Reference Position=453384; /Consensus Position=453244; /Reference=C;

/Variation Type=SNP; /Allele Variations=A; /Frequencies=100.0; /Counts=34;

/Coverage=34; /Overlapping Annotations=**Gene: ycsA**, CDS: ycsA; /Amino Acid

Change=**Ser185Arg**

**655624** /Reference Position=675995; /Consensus Position=655624; /Reference=A;

/Variation Type=SNP; /Allele Variations=G; /Frequencies=100.0; /Counts=46; /Coverage=46

**706584** /Reference Position=726955; /Consensus Position=706584; /Reference=C;

/Variation Type=SNP; /Allele Variations=A; /Frequencies=100.0; /Counts=62;

/Coverage=62; /Overlapping Annotations=**Gene: opuE**, CDS: opuE; /Amino Acid

Change=**Gly455Val**

**746223** /Reference Position=766594; /Consensus Position=746223; /Reference=A;

/Variation Type=SNP; /Allele Variations=G; /Frequencies=100.0; /Counts=81;

/Coverage=81; /Overlapping Annotations=**Gene: yesS**, CDS: yesS; /Amino Acid

Change=**Lys253Glu**

**914199** /Reference Position=934570; /Consensus Position=914199; /Reference=G;

/Variation Type=SNP; /Allele Variations=T; /Frequencies=100.0; /Counts=43; /Coverage=43;

/Overlapping Annotations=**Gene: yfhP**, CDS: yfhP; /Amino Acid Change=**Pro291Thr**

**1109581** /Reference Position=1129968; /Consensus Position=1109581; /Reference=T;

/Variation Type=SNP; /Allele Variations=C; /Frequencies=100.0; /Counts=65; /Coverage=65;

/Overlapping Annotations=**Gene: yhjM**, CDS: yhjM; /Amino Acid Change=**Val85Ala**

**1142226** /Reference Position=1162613; /Consensus Position=1142226; /Reference=T;

/Variation Type=SNP; /Allele Variations=C; /Frequencies=100.0; /Counts=45; /Coverage=45;

/Overlapping Annotations=**Gene: yisR**, CDS: yisR; /Amino Acid Change=**Leu116Pro**

**1158255** /Reference Position=1178642; /Consensus Position=1158255; /Reference=A;

/Variation Type=SNP; /Allele Variations=G; /Frequencies=100.0; /Counts=54;

/Coverage=54; /Overlapping Annotations=**Gene: yitI**, CDS: yitI; /Amino Acid

Change=**Val14Ala**

**1202445** /Reference Position=1222831; /Consensus Position=1202445; /Reference=G;

/Variation Type=Complex SNP; /Allele Variations=G/A; /Frequencies=62.3/37.7;

/Counts=33/20; /Coverage=53; /Overlapping Annotations=**Gene: oppC**, CDS: oppC; /Amino

Acid Change=**Trp100***

**1204138** /Reference Position=1224524; /Consensus Position=1204138; /Reference=T;

/Variation Type=SNP; /Allele Variations=G; /Frequencies=100.0; /Counts=101;

/Coverage=101; /Overlapping Annotations=**Gene: oppD**, CDS: oppD; /Amino Acid

Change=**Val357Gly**

**1243898** /Reference Position=1264284; /Consensus Position=1243898; /Reference=T;

/Variation Type=SNP; /Allele Variations=A; /Frequencies=87.1; /Counts=27; /Coverage=31;

/Overlapping Annotations=**Gene: yjcM**, CDS: yjcM; /Amino Acid Change=**Lys216Asn**

**1247081** /Reference Position=1267467; /Consensus Position=1247081; /Reference=G;

/Variation Type=Complex SNP; /Allele Variations=G/A; /Frequencies=51.7/48.3;

/Counts=46/43; /Coverage=89

**1247095** /Reference Position=1267481; /Consensus Position=1247095; /Reference=A;

/Variation Type=Complex SNP; /Allele Variations=A/T; /Frequencies=57.8/42.2;

/Counts=63/46; /Coverage=109

**1297292** /Reference Position=1317678; /Consensus Position=1297292; /Reference=G;

/Variation Type=Complex SNP; /Allele Variations=A/G; /Frequencies=54.3/45.7;

/Counts=57/48; /Coverage=105; /Overlapping Annotations=**Gene: xlyB**, CDS: xlyB

**1297295** /Reference Position=1317681; /Consensus Position=1297295; /Reference=C;

/Variation Type=Complex SNP; /Allele Variations=T/C; /Frequencies=53.5/46.5;

/Counts=54/47; /Coverage=101; /Overlapping Annotations=**Gene: xlyB**, CDS: xlyB

**1297298** /Reference Position=1317684; /Consensus Position=1297298; /Reference=A;

/Variation Type=Complex SNP; /Allele Variations=T/A; /Frequencies=56.7/43.3;

/Counts=55/42; /Coverage=97; /Overlapping Annotations=**Gene: xlyB**, CDS: xlyB

**1297322** /Reference Position=1317708; /Consensus Position=1297322; /Reference=G;

/Variation Type=Complex SNP; /Allele Variations=G/C; /Frequencies=65.0/35.0;

/Counts=39/21; /Coverage=60; /Overlapping Annotations=**Gene: xlyB**, CDS: xlyB

**1321822** /Reference Position=1342208; /Consensus Position=1321822; /Reference=T;

/Variation Type=SNP; /Allele Variations=C; /Frequencies=100.0; /Counts=53; /Coverage=53;

/Overlapping Annotations=**Gene: xkdT**, CDS: xkdT; /Amino Acid Change=**Phe287Leu**

**1352257** /Reference Position=1372643; /Consensus Position=1352257; /Reference=T;

/Variation Type=SNP; /Allele Variations=C; /Frequencies=100.0; /Counts=78; /Coverage=78

**1576862** /Reference Position=1597248; /Consensus Position=1576862; /Reference=C;

/Variation Type=SNP; /Allele Variations=T; /Frequencies=100.0; /Counts=74; /Coverage=74;

/Overlapping Annotations=**Gene: ftsA**, CDS: ftsA; /Amino Acid Change=**Leu259Phe**

**1643393** /Reference Position=1663779; /Consensus Position=1643393; /Reference=C;

/Variation Type=SNP; /Allele Variations=T; /Frequencies=100.0; /Counts=64; /Coverage=64;

/Overlapping Annotations=**Gene: fabD**, CDS: fabD

**1655463** /Reference Position=1675849; /Consensus Position=1655463; /Reference=C;

/Variation Type=SNP; /Allele Variations=T; /Frequencies=100.0; /Counts=67; /Coverage=67;

/Overlapping Annotations=**Gene: trmD**, CDS: trmD; /Amino Acid Change=**His227Tyr**

**1745974** /Reference Position=1766359; /Consensus Position=1745974; /Reference=C;

/Variation Type=SNP; /Allele Variations=T; /Frequencies=100.0; /Counts=60; /Coverage=60;

/Overlapping Annotations=**Gene: pbpX**, CDS: pbpX

**1761688** /Reference Position=1782073; /Consensus Position=1761688; /Reference=G;

/Variation Type=SNP; /Allele Variations=A; /Frequencies=100.0; /Counts=60;

/Coverage=60; /Overlapping Annotations=**Gene: pksA**, CDS: pksA

**1860156** /Reference Position=1880541; /Consensus Position=1860156; /Reference=T;

/Variation Type=Complex SNP; /Allele Variations=T/C; /Frequencies=57.1/42.9;

/Counts=44/33; /Coverage=77

**1860163** /Reference Position=1880548; /Consensus Position=1860163; /Reference=T;

/Variation Type=Complex SNP; /Allele Variations=T/G; /Frequencies=57.3/42.7;

/Counts=47/35; /Coverage=82

**1860165** /Reference Position=1880550; /Consensus Position=1860165; /Reference=T;

/Variation Type=Complex SNP; /Allele Variations=T/C; /Frequencies=57.0/43.0;

/Counts=45/34; /Coverage=79

**1860180** /Reference Position=1880565; /Consensus Position=1860180; /Reference=C;

/Variation Type=Complex SNP; /Allele Variations=C/T; /Frequencies=64.2/35.8;

/Counts=43/24; /Coverage=67

**1955774** /Reference Position=1976159; /Consensus Position=1955774; /Reference=C;

/Variation Type=SNP; /Allele Variations=T; /Frequencies=100.0; /Counts=39; /Coverage=39;

/Overlapping Annotations=**Gene: ppsC**, CDS: ppsC

**1990706** /Reference Position=2011091; /Consensus Position=1990706; /Reference=G;

/Variation Type=SNP; /Allele Variations=A; /Frequencies=100.0; /Counts=71;

/Coverage=71; /Overlapping Annotations=**Gene: gltA**, CDS: gltA; /Amino Acid

Change=**Ala1181Val**

**2020714** /Reference Position=2041099; /Consensus Position=2020714; /Reference=A;

/Variation Type=SNP; /Allele Variations=G; /Frequencies=100.0; /Counts=24;

/Coverage=24; /Overlapping Annotations=**Gene: yozT**, CDS: yozT

**2064511** /Reference Position=2084896; /Consensus Position=2064511; /Reference=G;

/Variation Type=Complex SNP; /Allele Variations=G/A; /Frequencies=60.0/40.0;

/Counts=3/2; /Coverage=5; /Overlapping Annotations=**Gene: czrA**, CDS: czrA; /Amino Acid

Change=**Leu72Phe**

**2079331** /Reference Position=2099716; /Consensus Position=2079331; /Reference=A;

/Variation Type=SNP; /Allele Variations=T; /Frequencies=100.0; /Counts=43; /Coverage=43;

/Overlapping Annotations=**Gene:yozO**, CDS: yozO

**2154053** /Reference Position=2174438; /Consensus Position=2154053; /Reference=T;

/Variation Type=SNP; /Allele Variations=C; /Frequencies=100.0; /Counts=92; /Coverage=92;

/Overlapping Annotations=Gene:**yorO**, CDS: yorO; /Amino Acid Change=**Arg38Gly**

**2181023** /Reference Position=2201408; /Consensus Position=2181023; /Reference=A;

/Variation Type=SNP; /Allele Variations=G; /Frequencies=100.0; /Counts=56;

/Coverage=56; /Overlapping Annotations=Gene:**yoqA**, CDS: yoqA; /Amino Acid

Change=**Leu23Pro**

**2195691** /Reference Position=2216076; /Consensus Position=2195691; /Reference=C;

/Variation Type=SNP; /Allele Variations=T; /Frequencies=100.0; /Counts=113;

/Coverage=113; /Overlapping Annotations=**Gene:yopA**, CDS: yopA; /Amino Acid

Change=**Trp234***

**2336854** /Reference Position=2357239; /Consensus Position=2336854; /Reference=C;

/Variation Type=Complex SNP; /Allele Variations=C/G; /Frequencies=60.0/40.0;

/Counts=3/2; /Coverage=5; /Overlapping Annotations=Gene:**ypjH**, CDS: ypjH; /Amino Acid

Change=**Val325Leu**

**2357296** /Reference Position=2377681; /Consensus Position=2357296; /Reference=A;

/Variation Type=SNP; /Allele Variations=G; /Frequencies=100.0; /Counts=56;

/Coverage=56; /Overlapping Annotations=**Gene:aroH**, CDS: aroH; /Amino Acid

Change=**Val112Ala**

**2382673^2382674** /Reference Position=2403062; /Consensus Position=2382674;

/Reference=C; /Variation Type=Complex SNP; /Allele Variations=C/G;

/Frequencies=58.3/41.7; /Counts=7/5; /Coverage=12; /Overlapping Annotations=**Gene:**

**gudB**, CDS: gudB; /Amino Acid Change=**Val97Leu**

**2382673^2382674** /Reference Position=2403064; /Consensus Position=2382674;

/Reference=G; /Variation Type=Complex SNP; /Allele Variations=T/G;

/Frequencies=55.6/44.4; /Counts=5/4; /Coverage=9; /Overlapping Annotations=**Gene: gudB**, CDS: gudB; /Amino Acid Change=**Ala96Glu**

**2401213** /Reference Position=2421606; /Consensus Position=2401213; /Reference=T;

/Variation Type=SNP; /Allele Variations=C; /Frequencies=100.0; /Counts=44; /Coverage=44;

/Overlapping Annotations=**Gene: rluB**, CDS: rluB

**2431322** /Reference Position=2451715; /Consensus Position=2431322; /Reference=G;

/Variation Type=SNP; /Allele Variations=A; /Frequencies=100.0; /Counts=21;

/Coverage=21; /Overlapping Annotations=**Gene:mleA**, CDS: mleA

**2460253** /Reference Position=2480646; /Consensus Position=2460253; /Reference=T;

/Variation Type=SNP; /Allele Variations=A; /Frequencies=85.7; /Counts=18; /Coverage=21

**2460254** /Reference Position=2480647; /Consensus Position=2460254; /Reference=A;

/Variation Type=SNP; /Allele Variations=T; /Frequencies=100.0; /Counts=18; /Coverage=18

**2460260^2460261** /Reference Position=2480654; /Consensus Position=2460261;

/Reference=T; /Variation Type=SNP; /Allele Variations=A; /Frequencies=75.0; /Counts=3;

/Coverage=4

**2460272^2460273** /Reference Position=2480667; /Consensus Position=2460273;

/Reference=T; /Variation Type=SNP; /Allele Variations=G; /Frequencies=100.0; /Counts=3;

/Coverage=3

**2640789** /Reference Position=2661184; /Consensus Position=2640789; /Reference=T;

/Variation Type=Complex SNP; /Allele Variations=T/C; /Frequencies=62.6/37.4;

/Counts=102/61; /Coverage=163; /Overlapping Annotations=**Gene: yqcG**, CDS: yqcG;

/Amino Acid Change=**Val28Ala**

**2640805** /Reference Position=2661200; /Consensus Position=2640805; /Reference=G;

/Variation Type=Complex SNP; /Allele Variations=G/A; /Frequencies=62.3/37.7;

/Counts=104/63; /Coverage=167; /Overlapping Annotations=**Gene: yqcG**, CDS: yqcG

**2640808** /Reference Position=2661203; /Consensus Position=2640808; /Reference=C;

/Variation Type=Complex SNP; /Allele Variations=C/T; /Frequencies=64.6/35.4;

/Counts=104/57; /Coverage=161; /Overlapping Annotations=**Gene: yqcG**, CDS: yqcG

**2640856** /Reference Position=2661251; /Consensus Position=2640856; /Reference=T;

/Variation Type=Complex SNP; /Allele Variations=T/C; /Frequencies=62.2/37.8;

/Counts=46/28; /Coverage=74; /Overlapping Annotations=**Gene: yqcG**, CDS: yqcG

**2640865** /Reference Position=2661260; /Consensus Position=2640865; /Reference=T;

/Variation Type=Complex SNP; /Allele Variations=T/C; /Frequencies=62.8/37.2;

/Counts=54/32; /Coverage=86; /Overlapping Annotations=**Gene: yqcG**, CDS: yqcG

**2640886** /Reference Position=2661281; /Consensus Position=2640886; /Reference=A;

/Variation Type=Complex SNP; /Allele Variations=A/G; /Frequencies=57.1/42.1;

/Counts=76/56; /Coverage=133; /Overlapping Annotations=**Gene: yqcG**, CDS: yqcG

**2640889** /Reference Position=2661284; /Consensus Position=2640889; /Reference=T;

/Variation Type=Complex SNP; /Allele Variations=T/C; /Frequencies=56.5/43.5;

/Counts=74/57; /Coverage=131; /Overlapping Annotations=**Gene: yqcG**, CDS: yqcG

**2640904** /Reference Position=2661299; /Consensus Position=2640904; /Reference=A;

/Variation Type=Complex SNP; /Allele Variations=G/A; /Frequencies=51.1/48.9;

/Counts=70/67; /Coverage=137; /Overlapping Annotations=**Gene: yqcG**, CDS: yqcG

**2640914** /Reference Position=2661309; /Consensus Position=2640914; /Reference=T;

/Variation Type=Complex SNP; /Allele Variations=C/T; /Frequencies=52.7/47.3;

/Counts=78/70; /Coverage=148; /Overlapping Annotations=**Gene: yqcG**, CDS: yqcG

**2640931** /Reference Position=2661326; /Consensus Position=2640931; /Reference=T;

/Variation Type=Complex SNP; /Allele Variations=A/T; /Frequencies=52.5/47.5;

/Counts=73/66; /Coverage=139; /Overlapping Annotations=**Gene: yqcG**, CDS: yqcG

**2640937** /Reference Position=2661332; /Consensus Position=2640937; /Reference=C;

/Variation Type=Complex SNP; /Allele Variations=T/C; /Frequencies=57.3/42.7;

/Counts=86/64; /Coverage=150; /Overlapping Annotations=**Gene: yqcG**, CDS: yqcG

**2640956..2640958** /Reference Position=2661351; /Consensus Position=2640956;

/Reference=TTT; /Variation Type=Complex SNP; /Allele Variations=ATC/TTT;

/Frequencies=55.4/44.0; /Counts=92/73; /Coverage=166; /Overlapping Annotations=CDS:

yqcG, **Gene: yqcG**; /Amino Acid Change=**Phe84Phe,Il**e

**2640961** /Reference Position=2661356; /Consensus Position=2640961; /Reference=A;

/Variation Type=Complex SNP; /Allele Variations=G/A; /Frequencies=55.9/44.1;

/Counts=104/82; /Coverage=186; /Overlapping Annotations=**Gene: yqcG**, CDS: yqcG

**2640972** /Reference Position=2661367; /Consensus Position=2640972; /Reference=A;

/Variation Type=Complex SNP; /Allele Variations=G/A; /Frequencies=59.1/40.9;

/Counts=97/67; /Coverage=164; /Overlapping Annotations=**Gene: yqcG**, CDS: yqcG;

/Amino Acid Change=**Asn89Ser**

**2641324** /Reference Position=2661719; /Consensus Position=2641324; /Reference=G;

/Variation Type=Complex SNP; /Allele Variations=G/C; /Frequencies=62.5/37.5;

/Counts=70/42; /Coverage=112; /Overlapping Annotations=**Gene: yqcG**, CDS: yqcG

**2641345** /Reference Position=2661740; /Consensus Position=2641345; /Reference=G;

/Variation Type=Complex SNP; /Allele Variations=G/A; /Frequencies=60.7/39.3;

/Counts=85/55; /Coverage=140; /Overlapping Annotations=**Gene: yqcG**, CDS: yqcG

**2641348** /Reference Position=2661743; /Consensus Position=2641348; /Reference=T;

/Variation Type=Complex SNP; /Allele Variations=T/C; /Frequencies=60.3/39.7;

/Counts=85/56; /Coverage=141; /Overlapping Annotations=**Gene: yqcG**, CDS: yqcG

**2641485** /Reference Position=2661880; /Consensus Position=2641485; /Reference=C;

/Variation Type=Complex SNP; /Allele Variations=C/T; /Frequencies=62.1/37.9;

/Counts=72/44; /Coverage=116; /Overlapping Annotations=**Gene: yqcG**, CDS: yqcG;

/Amino Acid Change=**Ala260Val**

**2641504** /Reference Position=2661899; /Consensus Position=2641504; /Reference=A;

/Variation Type=Complex SNP; /Allele Variations=A/G; /Frequencies=53.9/46.1;

/Counts=76/65; /Coverage=141; /Overlapping Annotations=**Gene: yqcG**, CDS: yqcG

**2641507** /Reference Position=2661902; /Consensus Position=2641507; /Reference=G;

/Variation Type=Complex SNP; /Allele Variations=G/A; /Frequencies=58.1/41.9;

/Counts=79/57; /Coverage=136; /Overlapping Annotations=**Gene: yqcG**, CDS: yqcG

**2641510** /Reference Position=2661905; /Consensus Position=2641510; /Reference=C;

/Variation Type=Complex SNP; /Allele Variations=C/A; /Frequencies=55.9/44.1;

/Counts=71/56; /Coverage=127; /Overlapping Annotations=**Gene: yqcG**, CDS: yqcG

**2641747** /Reference Position=2662142; /Consensus Position=2641747; /Reference=C;

/Variation Type=Complex SNP; /Allele Variations=C/T; /Frequencies=60.4/39.6;

/Counts=67/44; /Coverage=111; /Overlapping Annotations=**Gene: yqcG**, CDS: yqcG

/Variation Type=Complex SNP; /Allele Variations=T/C; /Frequencies=61.9/38.1;

/Counts=65/40; /Coverage=105; /Overlapping Annotations=**Gene: yqcG**, CDS: yqcG

**2641777** /Reference Position=2662172; /Consensus Position=2641777; /Reference=A;

/Variation Type=Complex SNP; /Allele Variations=A/T; /Frequencies=60.8/39.2;

/Counts=62/40; /Coverage=102; /Overlapping Annotations=**Gene: yqcG**, CDS: yqcG

**2644568** /Reference Position=2664963; /Consensus Position=2644568; /Reference=G;

/Variation Type=Complex SNP; /Allele Variations=G/A; /Frequencies=52.6/47.4;

/Counts=30/27; /Coverage=57; /Overlapping Annotations=**Gene: cwlA**, CDS: cwlA

**2644586** /Reference Position=2664981; /Consensus Position=2644586; /Reference=A;

/Variation Type=Complex SNP; /Allele Variations=A/G; /Frequencies=62.2/37.8;

/Counts=46/28; /Coverage=74; /Overlapping Annotations=**Gene: cwlA**, CDS: cwlA

**2644589** /Reference Position=2664984; /Consensus Position=2644589; /Reference=A;

/Variation Type=Complex SNP; /Allele Variations=A/G; /Frequencies=57.0/43.0;

/Counts=49/37; /Coverage=86; /Overlapping Annotations=**Gene: cwlA**, CDS: cwlA

**2644601** /Reference Position=2664996; /Consensus Position=2644601; /Reference=A;

/Variation Type=Complex SNP; /Allele Variations=G/A; /Frequencies=51.4/48.6;

/Counts=54/51; /Coverage=105; /Overlapping Annotations=**Gene: cwlA**, CDS: cwlA

**2644613** /Reference Position=2665008; /Consensus Position=2644613; /Reference=T;

/Variation Type=Complex SNP; /Allele Variations=T/A; /Frequencies=55.1/44.9;

/Counts=65/53; /Coverage=118; /Overlapping Annotations=**Gene: cwlA**, CDS: cwlA

**2709948** /Reference Position=2730342; /Consensus Position=2709948; /Reference=A;

/Variation Type=SNP; /Allele Variations=G; /Frequencies=100.0; /Counts=32; /Coverage=32

**2841739** /Reference Position=2862132; /Consensus Position=2841739; /Reference=A;

/Variation Type=Complex SNP; /Allele Variations=A/G; /Frequencies=52.1/47.9;

/Counts=25/23; /Coverage=48; /Overlapping Annotations=**Gene: radC**, CDS: radC; /Amino

Acid Change=**Leu135Ser**

**2841741** /Reference Position=2862134; /Consensus Position=2841741; /Reference=A;

/Variation Type=SNP; /Allele Variations=G; /Frequencies=77.4; /Counts=24; /Coverage=31;

/Overlapping Annotations=**Gene: radC**, CDS: radC

**2873513** /Reference Position=2893906; /Consensus Position=2873513; /Reference=G;

/Variation Type=SNP; /Allele Variations=A; /Frequencies=100.0; /Counts=90;

/Coverage=90; /Overlapping Annotations=**Gene: ilvC**, CDS: ilvC

**2962025** /Reference Position=2982417; /Consensus Position=2962025; /Reference=T;

/Variation Type=SNP; /Allele Variations=A; /Frequencies=100.0; /Counts=77; /Coverage=77

**2962045** /Reference Position=2982437; /Consensus Position=2962045; /Reference=T;

/Variation Type=SNP; /Allele Variations=C; /Frequencies=100.0; /Counts=52; /Coverage=52

/Variation Type=SNP; /Allele Variations=A; /Frequencies=100.0; /Counts=68; /Coverage=68

**3051806** /Reference Position=3072198; /Consensus Position=3051806; /Reference=C;

/Variation Type=SNP; /Allele Variations=T; /Frequencies=100.0; /Counts=79; /Coverage=79

**3078981** /Reference Position=3099373; /Consensus Position=3078981; /Reference=A;

/Variation Type=SNP; /Allele Variations=T; /Frequencies=100.0; /Counts=43; /Coverage=43;

/Overlapping Annotations=**Gene: amyD**, CDS: amyD; /Amino Acid Change=**His84Leu**

**3235064** /Reference Position=3255469; /Consensus Position=3235064; /Reference=A;

/Variation Type=SNP; /Allele Variations=C; /Frequencies=98.4; /Counts=63; /Coverage=64;

/Overlapping Annotations=**Gene: comP**, CDS: comP; /Amino Acid Change=**Phe124Val**

**3235099** /Reference Position=3255504; /Consensus Position=3235099; /Reference=C;

/Variation Type=SNP; /Allele Variations=T; /Frequencies=100.0; /Counts=51; /Coverage=51;

/Overlapping Annotations=**Gene: comP**, CDS: comP; /Amino Acid Change=**Arg112Lys**

**3236912** /Reference Position=3257317; /Consensus Position=3236912; /Reference=G;

/Variation Type=SNP; /Allele Variations=A; /Frequencies=100.0; /Counts=70; /Coverage=70

**3371271** /Reference Position=3391676; /Consensus Position=3371271; /Reference=A;

/Variation Type=SNP; /Allele Variations=G; /Frequencies=100.0; /Counts=57;

/Coverage=57; /Overlapping Annotations=**Gene: gerAA**, CDS: gerAA; /Amino Acid

Change=**Thr299Ala**

**3371508** /Reference Position=3391913; /Consensus Position=3371508; /Reference=T;

/Variation Type=SNP; /Allele Variations=A; /Frequencies=100.0; /Counts=50;

/Coverage=50; /Overlapping Annotations=**Gene: gerAA**, CDS: gerAA; /Amino Acid

Change=**Leu378Met**

**3574882** /Reference Position=3595286; /Consensus Position=3574882; /Reference=G;

/Variation Type=SNP; /Allele Variations=A; /Frequencies=100.0; /Counts=92; /Coverage=92

**3620060** /Reference Position=3640465; /Consensus Position=3620060; /Reference=T;

/Variation Type=SNP; /Allele Variations=C; /Frequencies=100.0; /Counts=44; /Coverage=44;

/Overlapping Annotations=**Gene: flgM**, CDS: flgM

**3638005** /Reference Position=3658410; /Consensus Position=3638005; /Reference=A;

/Variation Type=Complex SNP; /Allele Variations=A/C; /Frequencies=53.4/46.6;

/Counts=39/34; /Coverage=73; /Overlapping Annotations=**Gene: tuaA**,

**3676464** /Reference Position=3696869; /Consensus Position=3676464; /Reference=T;

/Variation Type=SNP; /Allele Variations=C; /Frequencies=100.0; /Counts=58; /Coverage=58;

/Overlapping Annotations=**Gene: pgdS**, CDS: pgdS

**3681066..3681068** /Reference Position=3701471; /Consensus Position=3681066;

/Reference=AAC; /Variation Type=Complex SNP; /Allele Variations=GTC;

/Frequencies=100.0; /Counts=58; /Coverage=58; /Overlapping Annotations=CDS:

rbsR, **Gene: rbsR**; /Amino Acid Change=**Asn21Val**

**3681481** /Reference Position=3701886; /Consensus Position=3681481; /Reference=T;

/Variation Type=SNP; /Allele Variations=G; /Frequencies=100.0; /Counts=55;

/Coverage=55; /Overlapping Annotations=**Gene: rbsR**, CDS: rbsR; /Amino Acid

Change=**Val159Gly**

**3801144** /Reference Position=3821549; /Consensus Position=3801144; /Reference=T;

/Variation Type=SNP; /Allele Variations=A; /Frequencies=100.0; /Counts=46; /Coverage=46

**3881901** /Reference Position=3902306; /Consensus Position=3881901; /Reference=C;

/Variation Type=SNP; /Allele Variations=A; /Frequencies=100.0; /Counts=119;

/Coverage=119; /Overlapping Annotations=**Gene: sacA**, CDS: sacA; /Amino Acid

Change=**Leu448Phe**

**3953514** /Reference Position=3973920; /Consensus Position=3953514; /Reference=G;

/Variation Type=SNP; /Allele Variations=A; /Frequencies=100.0; /Counts=51;

/Coverage=51; /Overlapping Annotations=**Gene: cydD**, CDS: cydD; /Amino Acid

Change=**Ser391Phe**

**3975328** /Reference Position=3995734; /Consensus Position=3975328; /Reference=T;

/Variation Type=SNP; /Allele Variations=C; /Frequencies=100.0; /Counts=92; /Coverage=92;

/Overlapping Annotations=**Gene: pepT**, CDS: pepT

**4075404** /Reference Position=4095811; /Consensus Position=4075404; /Reference=C;

/Variation Type=SNP; /Allele Variations=T; /Frequencies=100.0; /Counts=72; /Coverage=72;

/Overlapping Annotations=**Gene: yxbD**, CDS: yxbD; /Amino Acid Change=**Val9Ile**
