# Supplementary material for: Genome analysis of a Bacillus subtilis strain reveals genetic mutations determining biocontrol properties
Source: World J Microbiol Biotechnol. 2019 Mar 13;35(3):52. doi: 10.1007/s11274-019-2625-x (PMC6435635; doi:10.1007/s11274-019-2625-x)
Supplement: Supplementary file 3 — Online Resource 3. Deletion/insertion type variants in the genome of Bacillus subtilis SZMC 6179J. (DOCX 14 KB) [file 11274_2019_2625_MOESM3_ESM.docx]

**Online Resource 3.** Deletion/insertion type variants in the genome of *Bacillus subtilis* SZMC 6179J

| **Gene** | **Position and changes** | **Name and function of the protein product** |
| --- | --- | --- |
| *srf*AC | **399063^399064** /Reference Position=399196; /Consensus Position=399064; /Reference=AAA; /Variation Type=DIP; /Allele Variations=---;  Amino Acid Change=Gln222_Lys223del insGln | surfactin synthase subunit 3; probably activates a leucine |
| *sfp* | **407532^407533** /Reference Position=407668; /Consensus Position=407533; /Reference=T; /Variation Type=DIP; /Allele Variations=-; | 4'-phosphopantetheinyl transferase; activates the seven peptidyl carrier protein (PCP) domains of surfactin synthase SRF1/2/3 by transferring the 4'-phosphopantetheinyl moiety of coenzyme A (CoA) to a serine residue; required for cells of *B. subtilis* to become producers of the lipopeptide antibiotics surfactin and fengycin |
| *yhg*B | **1062764** /Reference Position=1083152; /Consensus Position=1062764; /Reference=-; /Variation Type=DIP; /Allele Variations=C;  Amino Acid Change=Asp12fs | uncharacterized protein |
| *app*A | **1193611** /Reference Position=1213998; /Consensus Position=1193611; /Reference=-; /Variation Type=Complex DIP; /Allele Variations=A | oligopeptide-binding protein AppA; a component of an oligopeptide permease, a binding protein-dependent transport system |
| *ymf*K | **1740904..1740905** /Reference Position=1761291; /Consensus Position=1740904; /Reference=--; /Variation Type=Complex DIP; /Allele Variations=GT | uncharacterized protein |
| *gud*B | **2382674** /Reference Position=2403059; /Consensus Position=2382674; /Reference=TCACCGCCT; /Variation Type=DIP; /Allele Variations=---------;  Amino Acid Change=Lys95_Lys98 del insLys | cryptic catabolic NAD-specific  glutamate dehydrogenase GudB |
| *yrk*H | **2691802** /Reference Position=2712197; /Consensus Position=2691802; /Reference=-; /Variation Type=Complex DIP; /Allele Variations=-/T; /  Amino Acid Change=Lys16fs | uncharacterized protein |
| *swr*AA | **3601538^3601539** /Reference Position=3621943; /Consensus Position=3601539; /Reference=T; /Variation Type=Complex DIP; /Allele Variations=-/T; | swarming motility protein SwrAA, required for swarm cell differentiation, plays a crucial role in regulating the degree of cell flagellation |
| *ywb*D | **3915418^3915419** /Reference Position=3935824; /Consensus Position=3915419; /Reference=T; /Variation Type=DIP  Amino Acid Change=*397fs | putative ribosomal RNA large subunit methyltransferase |
